# Surgical Multidisciplinary Team Meetings Are Enhanced by Collaboration in the Metaverse

**DOI:** 10.1089/jmxr.2023.0004

**Published:** 2024-06-10

**Authors:** Rojan Ghaderi, Thomas Edwards, Justin Cobb, Kartik Logishetty

**Affiliations:** MSk Lab, Department of Surgery and Cancer, Faculty of Medicine, Imperial College London, London, United Kingdom.

**Keywords:** Multidisciplinary team, Virtual Reality, Teamwork, Collaboration, Patient centred care

## Abstract

Multidisciplinary team meetings (MDT-Ms) are utilized as a tool for clinical decision-making. Currently, these are in-person or remotely attended via screen-sharing of patients’ electronic health records, including clinical findings, numerical data, and two-dimensional and three-dimensional imaging. Immersive virtual reality (IVR) is an emerging technology that allows multiple care-providers to remotely interact with each other and with patient data in a metaverse—an immersive and collaborative digital environment. An MDT-M held in the metaverse could enhance surgical decision-making. To develop an IVR metaverse for MDT-M and to test its feasibility and efficacy to visualize data and make clinical decisions in the orthopedic trauma setting. Interviews of users attending existing orthopedic trauma MDT-Ms at a regional major trauma center identified strengths, weaknesses, opportunities, and threats for a novel metaverse platform. This was iteratively tested for feature development, and 24 simulated cases were curated from previous admissions. Pilot MDT-Ms were conducted, and user experience was measured using the NASA Task Load Index (NASA-TLX) and System Usability Scale. An IVR metaverse platform was developed to conduct MDT-Ms with multiple remote participants. Four MDT-Ms were successfully conducted with health care professionals (*n* = 13). Participants found that “data visualization” and “clinical decision-making” were effective in IVR: median 5 (interquartile range [IQR], 4–5) and 5 (IQR, 4–5), respectively. Ratings on “ease of communication” in the metaverse varied with a median of 3 (IQR, 2–4.5). Qualitative analysis highlighted the strength of IVR to interrogate 3D anatomy and injury patterns collaboratively and quickly. The NASA-TLX domain “task performance” was rated consistently highly, indicating the ability to use presented data to advance patient management. Health care professionals can feasibly conduct complex MDT-Ms using a novel metaverse platform on commercially available IVR headsets. Participants were able to collaboratively interrogate clinical data, facilitating enhanced discussion, decision-making, and perceived task performance. Future iterations will focus on improving the designation of roles to improve communication in the metaverse meeting.

## Background

A multidisciplinary team (MDT) in health care is defined as a group of individuals across disciplines, who facilitate holistic decision-making related to patient care.^1^ The MDT meeting (MDT-M) involves discussion of complex cases by health care professionals, with the aim of improving diagnosis and creating a detailed management plan specific to the individual clinical and psychosocial needs of the patient.^2,3^ Studies analyzing the perspectives of MDT members have found that scheduling conflicts owing to other responsibilities, poor cooperation within the team, and challenges in viewing multiple information sources at once are some of the factors that hinder effective MDT-Ms.^4,5^

The rise of hybrid virtual working during the COVID-19 pandemic has spurred a drive toward digitalization in health care.^6,7^ In immersive virtual reality (IVR), users utilize head-mounted displays to view a completely virtual environment. The ability of IVR to display accurate three-dimensional (3D) models of patient anatomy lends itself to use as a surgical planning tool.^8^ IVR has enhanced teamwork in training of nurses and for planning neurosurgery.^8,9^ Interprofessional education via IVR can deliver a significant increase in collaborative attitudes.^10^ In surgical training, IVR can enhance knowledge and technical skills of surgeons^11^ and nurses.^12^ Multiplayer IVR can enhance interdisciplinary, nontechnical skills, including communication, leadership, and decision-making.^13^

Extended reality (XR) technology is a unifying immersive and collaborative digital environment, in which users can interact with each other and their surroundings in a life-like manner and outside of a defined timeline,^6,14^ for a specific experience, or to perform specific tasks.^14^ XR technology designed for conducting MDT-Ms in health care could address challenges of existing in-person MDTs while enhancing collaboration and decision-making.^15^

Orthopedic trauma MDT-Ms are typically attended by surgeons of varying subspecialties, nurses, physiotherapists, radiologists, microbiologists, and physicians. They rely on health care professionals evaluating clinical findings, laboratory results, and multimodal imaging to create individualized management plans.^16–18^ Imaging typically includes clinical photographs, plain radiographs, and multiplanar images from ultrasound, computed tomography (CT), and magnetic resonance imaging and 3D reconstructions. Management requires an understanding of patient factors, injury factors, local system factors, and cost-effectiveness.^19^ There is a wide demographic of patients presenting with orthopedic trauma, including elderly patients with fragility fractures and typically younger patients with high-energy trauma to multiple regions of the body. This calls for patient-centered conservative and surgical management plans^19,20^ and timely decision-making to determine priority and urgency.^21^ Orthopedic trauma surgery is thus an ideal situation for testing whether an XR technology can better facilitate patient-centered decision-making.

Our aim is to develop an IVR-based technology for a surgical MDT-M and to pilot its feasibility and efficacy to visualize data and make clinical decisions in the orthopedic trauma setting.

## Methods

### Study design

After institutional approval, this study was conducted in four stages: (1) qualitative evaluation of existing orthopedic trauma MDTs, (2) a scoping review of the literature and the commercial market for existing XR technology solutions, (3) development of an XR technology MDT-M, and (4) pilot testing with multiple health care professionals conducting an XR technology MDT-M using simulated orthopedic trauma and planned surgical cases.

#### Evaluation of existing orthopedic trauma MDT-Ms

Semi-structured interviews were conducted with health care professionals attending four consecutive orthopedic trauma MDT-Ms at a regional tertiary trauma center, collating their opinions on literature-identified barriers and facilitators of MDT-Ms. To develop a feature list for a minimum viable product XR platform, the MDT-Ms were evaluated to determine what patient data are required, what software is currently in use to present clinical data, the format of the MDT-M (whether entirely in-person, online, or hybrid), and location and role of attendees. A SWOT (strengths, weaknesses, opportunities, and threats) analysis was conducted with these data. Finally, the clinical pathway of 50 consecutive patients presenting to the orthopedic department was evaluated to create decision trees. This would allow for the MDT members to be asked standardized questions and systematically record their decisions for each patient, akin to a chatbot.^22^

#### Scoping review of the literature and testing of commercially available XR technology products

A scoping review of relevant literature was performed by searching PubMed Medical Literature Analysis and Retrieval System Online and Cumulative Index to Nursing and Allied Health Literature from inception through August 2022. The keywords were “virtual reality,” “MDT” or “multidisciplinary team,” and “clinical decision.” This included all medical and surgical specialties. Results were screened for relevance to the study. The findings of remaining studies were evaluated for existing applications of IVR in MDT-Ms and the respective challenges and facilitators to their implementation.

Attendance at digital health technology conferences and exhibitions (Future Surgery, London, and Giant Health, London) between February and December 2022 identified existing XR technology solutions for medical education and training. This provided an understanding of health care innovations in MDT-Ms, previously identified challenges, and existing applications of IVR in this domain.

Commercially available software solutions were then evaluated for their suitability for conducting an MDT-M and closeness of fit to the minimum viable product feature list. The assumption was that it would be faster and more cost-effective to adapt an existing and widely available software than build one *ab initio*. Similar testing was carried out for hardware and devices, and the most appropriate hardware was selected based on a combination of suitability for study aims, practicality, and costs.

#### Developing and pilot testing an XR technology MDT-M

##### Development

Information Centric Engineering (ICE) principles were used to adapt an existing, commercially available IVR platform so that it could deliver an MDT-M suitable for multiple health care professionals collaborating to make decisions for patients with complex orthopedic trauma. ICE involves modeling, simulation, and exchange.^23,24^ Modeling, simulation, and exchange (of information) are the three core facets of ICE. Modeling involves the creation of functional and process models that describe the target simulator in an information-intensive document, including the resources and technologies required to deliver it. Simulation involves the design of 3D simulation models (in IVR) for processes at various levels of abstraction. The third facet, exchange, involves the collaborative exchange of information and data between cross-functional users so that ideas and models are communicated in real time to enable timely assessment, comparison, and validation of candidate plans. This can involve face-to-face interactions typically between subject matter experts (in this case, orthopedic surgeons) and software engineers, written communications, and cyber–physical interactions for collaborative development within virtual reality spaces. This latter approach enables team members who are geographically distributed to interact within a virtual space to develop IVR solutions in an agile way. “Agile software development”^25^ refers to a collaborative approach between designers with different technical know-how, performed with the following principles in mind: (1) early and continuous delivery, (2) welcome of changing requirements, (3) short iterations and releases, (4) motivated individuals, (5) working software as a measure of progress, (6) technical excellence, (7) simplicity, and (8) self-organizing and reflective teams.

Data from the qualitative evaluation of existing orthopedic trauma MDT-Ms were used as the ground truth for adapting a virtual environment. Software programmers collaborated with orthopedic surgeons to iteratively evaluate mock patient datasets, including electronic health record (her) data, digital imaging and communications in medicine (DICOM) data, and management options to create an MDT-M XR technology with clinical relevance, technical feasibility, and face validity. The minimum viable product was developed and implemented through videoconferences and email correspondence. There were user tests, bug tracking, and iterative software releases. ICE and agile development resulted in a reduction in time to design and build the simulator environment, a reduction in the number of changes required between collaborative sessions, and a better understanding of the complexities of the simulation by all team members.

### Medicalholodeck^®^ platform

**FIG. 1.**
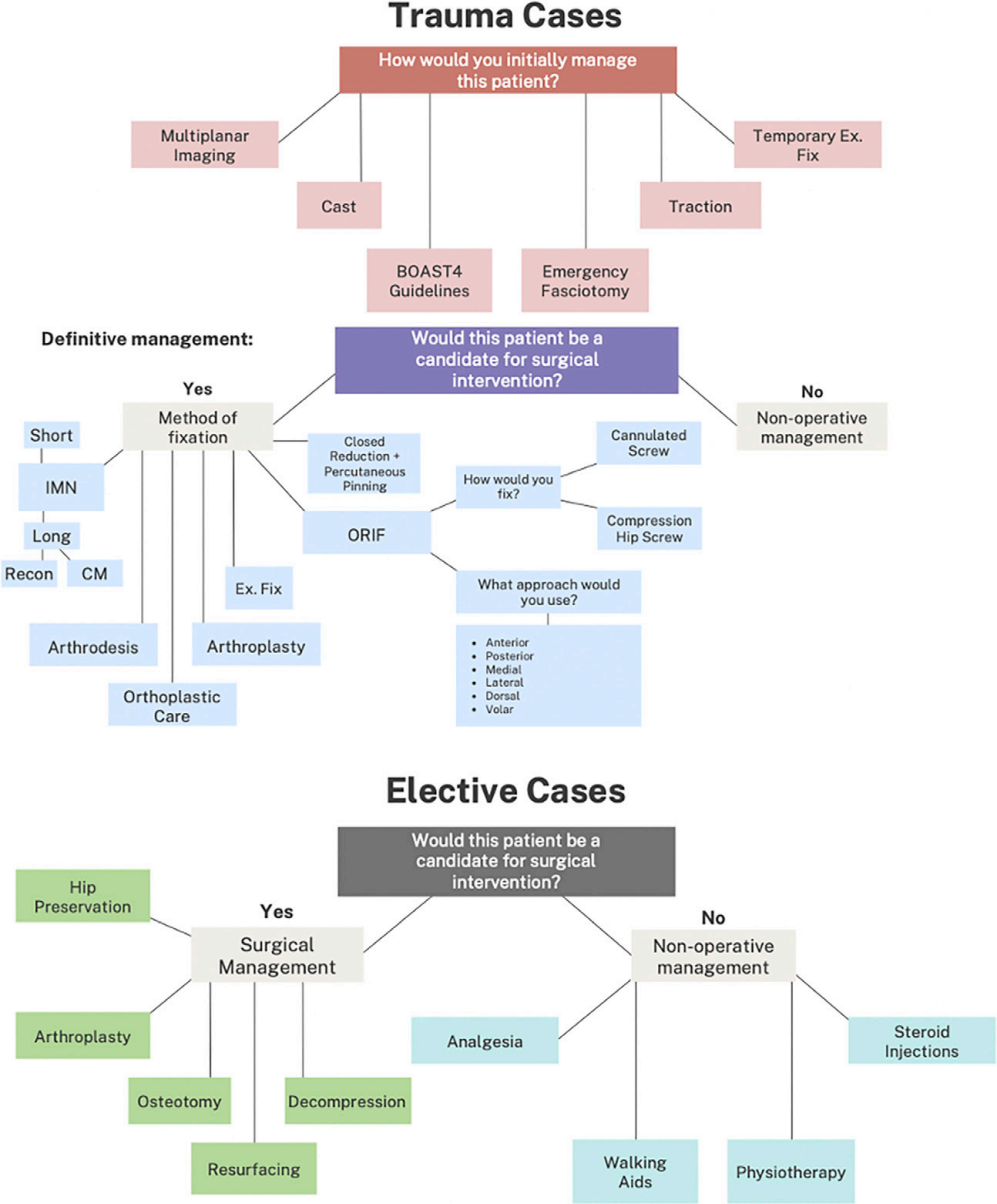
Clinical decision trees derived to facilitate structured discussion of patient cases and to record final management decisions. BOAST, British Orthopaedic Association Standards for Trauma; CM, cephalomedullary; Ex. Fix, external fixation; IMN, intramedullary nail; ORIF, Open Reduction and Internal Fixation.

**FIG. 2.**
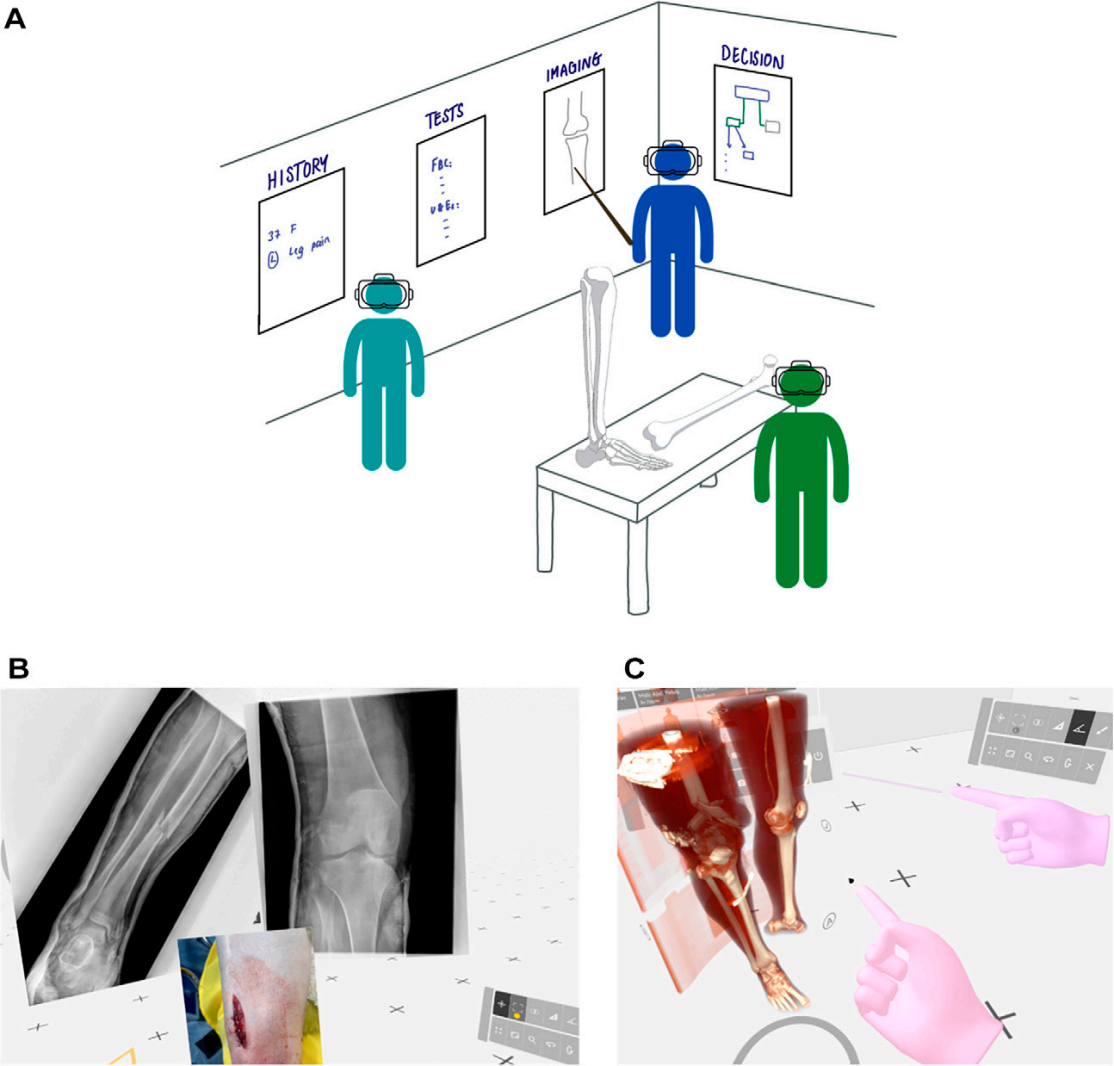
Depictions of the IVR MDT-M platform and setup. **(A)** Visual representation of the initial format intended for IVR MDT-M environment. Participants are depicted with headsets and viewing patient cases on surrounding panels, entitled history, tests, imaging, and decisions. **(B)** and **(C)** Images displaying the IVR MDT environment as seen in the pilot MDT. **(B)** Multimedia files in a trauma case (open tibial fracture), as seen in the Medicalholodeck^®^ IVR MDT environment. Photograph of the wound and radiographs of the tibia and knee are shown as seen in space. **(C)** Participants interacting with a three-dimensional reconstruction of a computed tomography scan depicting comminuted femur fracture, in IVR. The toolbar used by participants to interact with imaging is displayed in the image background. IVR, immersive virtual reality; MDT-M, multidisciplinary team meeting.

The virtual reality (VR) platform used to host the trial MDT in this study was Medicalholodeck, a software used for medical applications in VR. This existing software addressed several of the requirements of a VR MDT, as displayed in Figure 1. The base software consists of Medical Imaging XR, Dissection Master XR, and Anatomy Master XR. However, the commercially available solution did not allow for multiple datasets to be viewed simultaneously, did not allow for text files or photographs to be uploaded, and had basic multiplayer capability. Collaboration with Medicalholodeck led to the development of a bespoke, adapted platform with additional functionalities. The desired structure for the platform was conveyed as depicted in Figure 2a.

In this study, the Medical Imaging XR was used for viewing of the anonymized DICOM files in the simulated cases, as in Figures 2b and 2c. DICOMs were viewed in 3D format from the individual slices. Interaction with DICOMs included a slice function with the left hand, length measurement tool, angle measurement tool, drawing tool, and placement of arrow markers. Further functions included a zoom tool, movement of the DICOM, enlargement, and rotation in both *x* and *y* planes. The modality, contrast, and coloring of DICOMs were adjustable. DICOMs for each case were appropriately uploaded to laptops in advance and were uploaded to the platform via the “upload from drive” function.

#### Pilot testing

##### Participants and questionnaires

Consenting volunteer participants were recruited from existing memberships of two MDT-Ms, in orthopedic trauma, and hip and knee arthroplasty, based at Imperial College Healthcare NHS Trust. Participation required consent to participate in the pilot MDT and completion of questionnaires as given in Supplementary Data S1 and Supplementary Data S2. Exclusion criteria included a history of motion sickness, history of migraines, and history of epilepsy, because of the possibility of adverse effects to VR.^26^

##### Outcome measures

The meeting questionnaire in Supplementary Data S1 evaluated participants’ familiarity and experience with orthopedic MDT-Ms and IVR software. Meeting questionnaires in Supplementary Data S2 evaluated participant experiences of the XR MDT-M. Validated questionnaires of the NASA Task Load Index (NASA-TLX)^27^ and System Usability Scale (SUS)^28^ were adapted to the given task and incorporated into the questionnaire given in Supplementary Data S2. The NASA-TLX allows subjective evaluation of the demand involved in task completion across the following six domains: mental demand, temporal demand, performance, effort, frustration, and physical demand. It has been widely validated and used in the analysis of tasks, both in surgical specialties and IVR.^29,30^ SUS is used for evaluation of software and was adapted in this study to specifically focus on the XR MDT.^28^ Statements were rated from “Strongly Disagree” to “Strongly Agree” on a scale translating to a Likert scale (1–5).

The pilot tests of the XR technology MDT-M were evaluated using the following outcomes: number of cases discussed, average time taken for each case, and the proportion of cases in which the management plan was established. User experience outcomes evaluated users’ perceived opinions on the IVR MDT-M. These included perceptions on ease of use, case visualization, communication, task completion, and demand.

##### Simulated cases

Patient cases were compiled for discussion in the MDT trial. Anonymized DICOMs were identified from existing image banks at linked centers (Imperial College Healthcare NHS Trust and MSk Lab, Imperial College London), and mock cases were compiled around their presentation. Patients had consented for the use of their anonymized data for education and research. A total of 24 cases were prepared, of which 12 were trauma cases and 12 were elective cases. They included a diverse range of lower limb clinical cases and consisted of DICOMs, a summary of history and examination findings, and any relevant images. Cases were reviewed by two fellowship-trained orthopedic surgeons for clinical accuracy. A decision matrix was developed for recording decisions on initial and definitive management of both trauma and elective cases, as shown in Figure 1.

### Results

#### Evaluation of existing orthopedic trauma MDT-Ms

A total of 25 orthopedic trauma meeting members completed semi-structured interviews. There were three attending surgeons, two attending anesthesiologists, eight orthopedic residents, eight junior doctors, two medical students, one trauma nurse, and one trauma coordinator. This delivered the following three outputs: (1) a SWOT analysis of existing MDT-Ms (Table 1), (2) a feature list for a minimum viable product to conduct an MDT-M (Table 2), and (3) clinical decision trees (Fig. 1).

**Table 1.** SWOT Analysis of Existing Multidisciplinary Team Meetings in Orthopedics

Strengths	Microsoft Teams is widely accessible
	Screen-sharing allows unlimited range of information to be shared
	Can be used on desktop or mobile devices
Weaknesses	Information sharing is led by one operator
	All users see only the information as presented by the lead operator, in their chronology and (for imaging) view
	Frequent flips between windows showing different information can be disorientating
	Not personalizable
	Hierarchical, not promoting broader MDT-M members to contribute
	Limited educational value as MDT-M can become an unstructured discourse between experts
Opportunities	Interactivity with EHR without disrupting meeting
	Artificial intelligence–assisted decision-making
	Educational content creation from MDT-Ms
Threats	Balancing cost-effectiveness and innovation in public health care

EHR, electronic health record; MDT-M, multidisciplinary team meeting; SWOT, strengths, weaknesses, opportunities, and threats.

**Table 2.** Features for a Minimum Viable Product to Conduct a Surgical Multidisciplinary Team Meeting

Category	Feature
Connectivity	Remote and in-person capability
	Ability to view on mobile device
Access to information	DICOM viewer
	Photographic imaging viewer
	Laboratory data viewer
	PDF viewer
Ability to further patient management	Ability to record clinical decisions
	Ability to speak to other attendees

DICOM, digital imaging and communications in medicine; PDF, portable document format.

**Table 3.** Advantages and Disadvantages of Software and Hardware Options

	Advantages	Disadvantages
Hardware		
Oculus Rift S (Meta)	Accurate motion tracking—enables greater interactions in MDT	Tethered headsets—reliant on Alienware laptops and restricted movement
	Completely immersive—relevant to evaluation of IVR MDTs	High cost—require technical gaming laptop to run
Oculus Quest (Meta)	Untethered headsets—allow greater unrestricted range of movement in space	—
	High resolution of imaging	
	Low cost—increased accessibility and greater number of members	
Varjo XR-3 (Varjo)	Has both IVR and MR capabilities—relevant to testing of IVR MDT and allows overlaying of images onto real-world video	No applications for MR in this study—not relevant
	High resolution-greater visualization of imaging	
HTC Vive Pro 2.0 (HTC)	Completely immersive—relevant to evaluation of IVR MDTs	Tethered headsets—reliant on Alienware laptops and restricted in movement
	High resolution of imaging	
		High cost—require technical gaming laptop to run
HP Reverb G2 (HP)	Lightweight headset	PC-powered, tethered headset—restricted in movement
	High image resolution	High cost in addition to the cost of the technical gaming laptop
	Has both IVR and MR capabilities	No applications for MR in this study
PICO Neo 3 (ByteDance)	Untethered headsets allowing full range	Limited software options available—only via PICO store
Lenovo Mirage VR S3 (Lenovo)	Untethered headsets, with stand-alone VR capabilities High resolution of images	Low storage
Software		
MeetinVR	Distinct avatars allowing clear participant roles	Meeting room in virtual environment—imaging displayed in 2D and issues of resolution
		Development of virtual meeting room environment—funding and timeline considerations
Medicalholodeck*^®^*	3D visualization capabilities allowing appreciation of anatomical structures	Lacking functionalities: .pdf viewer, .jpeg/.png viewer, file organizer
	Designed for medical education-allows uploading of DICOMs	
Pixelmolkerei	Specializes in development of complex IVR surgical simulations—allows for detailed simulated environment	Simulations not relevant to the aim of this study on IVR MDTs
ImmersiveView	Team compatibility	Only DICOM compatibility, lacks .png/.jpeg
	Allows DICOM upload, combines scans as one unified 3D reconstruction	
MedicalImagingVR	Educational software enabling users to view DICOM reconstructions	Lacks functionalities including team capabilities, and other file types are not supported (.png/.jpeg, .pdf)
PrecisionOS	Software suitable for educational and pre-operative planning purposes	Lacking team compatibility
	Enables DICOM reconstruction	High fidelity simulation lacks relevance to this study
	High fidelity and resolution simulations	
	PACS integration	
True3D	PACS integration allows ease of DICOM upload	Specializes in 3D reconstruction of soft tissues
In-house (MSk lab) development	Flexibility of options and specialized features specific to MDT requirements	Timeline restrictions with development of required features

3D, three-dimensional; DICOM, digital imaging and communications in medicine; IVR, immersive virtual reality; MDT-M, multidisciplinary team meeting; MR, mixed reality.

#### Scoping review of the literature and testing of commercially available XR technology products

Scoping of the literature noted that 48 studies were found. Further screening limited this to 27 studies relevant to MDT applications of VR, of which 23 related to multidisciplinary surgical planning and 4 discussed multidisciplinary meetings. There were no instances of IVR MDT-Ms in surgical specialties. Key challenges were identified as image quality, access to IVR technology, and the security of platform utilized for discussion; facilitating factors included team interaction and the ability to accurately indicate lesions in a 3D space.

##### Outcomes

Investigated outcomes were separated into the following three categories: measures relating to feasibility, efficacy, and user experience. Feasibility was defined as the ability to complete an MDT clinical discussion of the given cases in IVR. This was measured by MDT completion and successful visualization of the simulated cases. Efficacy was evaluated as the quality of the clinical discussion.

##### Hardware

This study utilized five IVR headsets in total: three Oculus (Meta) Rift and two Oculus Quest headsets, all connected to high-speed internet. Headsets were selected based on institutional access and their advantages in the hardware analysis.

All headsets ran the adapted Medicalholodeck software version locally.

##### Pilot MDT

The pilot MDT consisted of cases presented in IVR by the MDT coordinator, composed of a patient case summary in .pdf format, CT scans and radiographs as DICOM files, and available clinical photographs in .jpeg format. CT scans were reconstructed into 3D visualization, and cases were discussed by participants with questions regarding patient compatibility with surgical intervention. Final management options were logged onto questionnaire version of the decision matrix in Figure 1.

##### Data processing

Data were exported into Microsoft Excel (version 16.61.1) and analyzed using GraphPad Prism (version 9.3.0), where they were presented graphically. Statistical analyses were conducted to test normality distribution of the data using Shapiro–Wilk test and quantile–quantile plots, yielding nonparametric data throughout. Mann–Whitney U test was used to compare the means between groups of nonparametric data. *p*-Values < 0.05 were considered as statistically significant in all analyses.

## Results

Data were collected across six IVR MDT-M sessions. In total, there were 13 (*n* = 13) participants, as summarized in Table 4. Most participants were surgeons (*n* = 8), and most reported having prior experience of XR (*n* = 11). The reported MDT experience varied, with “Sometimes (e.g., Several times a month)” being the most stated. The most common uses for XR were stated as being for education, research, and recreational purposes, in the order of decreasing frequency. Online MDTs were reported as the most common mode of MDT-Ms.

**Table 4.** Participant Characteristics of MDT Role and Experience

	No. of participants
Total participants	13
MDT role	
Medical student	1
Surgeon	8
Physician associate	1
Doctor	1
Engineer	2
MDT experience	
Daily	1
Regularly (multiple times a week)	3
Sometimes (several times a month)	6
Rarely (<1 a month)	3
Never	—
Prior XR experience	
Yes	11
No	2
XR experience	
Daily	—
Regularly (multiple times a week)	1
Sometimes (several times a month)	4
Rarely (<1 a month)	6
Never	2
Purpose(s) of XR use	
Education	8
Research	3
Work	1
Recreational	3

Table displaying participant (*n* = 13) characteristics in terms of MDT role and experience and XR experience and use. Sections with no participants are represented with —. MDT, multidisciplinary team; XR, extended reality.

**Table 5.** Results of the System Usability Scale

	Median	Interquartile range	Interpretation of median
I thought that it was easy to communicate with other MDT members in the VR environment	3	(2–4.5)	Neither Agree Nor Disagree
I was able to successfully view patient imaging	5	(4–5)	Strongly Agree
I was able to make clinical decisions based on how the information was presented in VR	5	(4–5)	Strongly Agree
I needed to learn a lot of things before using this VR platform	2	(1–4.5)	Somewhat Disagree
I thought that the data (2D imaging, 3D imaging, text) were well integrated	4	(3.5–4.5)	Somewhat Agree
I think that I would need the support of a technical person to use this VR platform	3	(2–5)	Neither Agree Nor Disagree
I thought that the platform was easy to use	3	(2–4)	Neither Agree Nor Disagree
I think that I would like to use a VR platform for MDT meetings regularly	4	(2–5)	Somewhat Agree

Participants rated the given statements, and numerical values were given to the scores: *Strongly Disagree* (1), *Somewhat Disagree* (2), *Neither Agree Nor Disagree* (3), *Somewhat Agree* (4), *Strongly Agree* (5). Median, interpretation of median, and interquartile range values have been given. 2D, two-dimensional; 3D, three-dimensional; MDT, multidisciplinary team; VR, virtual reality.

### Feasibility outcomes

Multiple MDT-Ms were successfully conducted in IVR, and simulated patient cases were successfully visualized. Connectivity issues were reported by multiple participants on a tethered Oculus Rift headset. Management decisions were logged by a coordinating member out of IVR.

### Efficacy outcomes

A total of 16 cases were discussed, across 5 MDT sessions, with a mean time taken per case of 7.8 min (standard deviation = 6.1). All cases (16/16) resulted in management plans.

### User experience

**FIG. 3.**
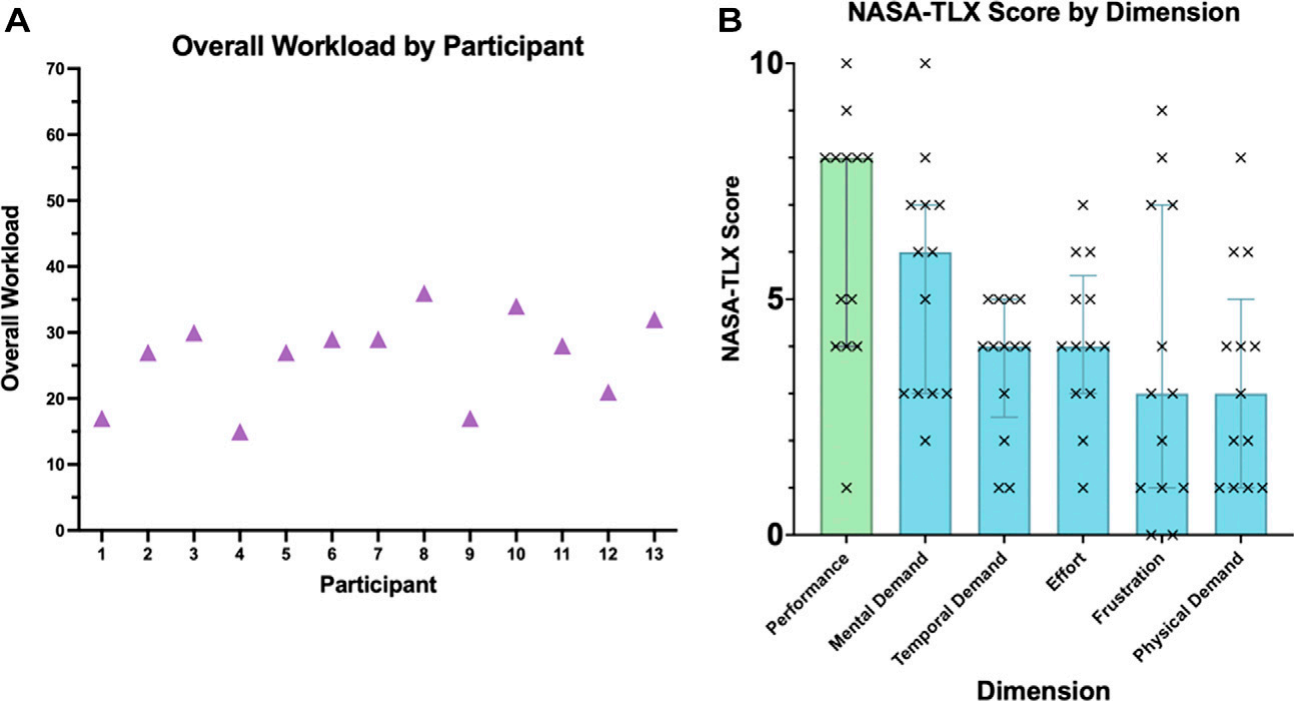
Results of the NASA-TLX displayed by overall workload and individual dimensions. **(A)** Scatter plot of the overall workload on the NASA-TLX. Overall workload for an individual participant is calculated by combining values of NASA-TLX score for each dimension. The given line displays the median value of 27 (IQR, 16–26.5). **(B)** Bar chart depicting the median values for NASA-TLX scores by dimension, and error bars represent the IQR. Participant scores are plotted as individual data points. Positive dimensions are shown in green, with negative dimensions in blue. Dimensions are shown in order of decreasing median values. IQR, interquartile range; NASA-TLX, NASA Task Load Index.

Participant scores on the NASA-TLX yielded overall workload scores and scores by dimension, as shown in Figures 3a and 3b, respectively. Overall workload had a median value of 27 (interquartile range [IQR], 16.0–28.5). By dimension, performance was the highest rated metric with a median of 8.0 (IQR, 4.0–8.0). Physical demand and frustration were rated the lowest and had median scores of 3.0 (IQR, 1.0–5.0) and 3.0 (IQR, 1.0–7.0), respectively.

## Discussion

The principal finding of this study was that IVR was both feasible and effective as a method of delivering MDT-Ms. These early data revealed several potential advantages to this approach, most notably, ease of information delivery, and high-fidelity imaging manipulation. This appears to enhance the ability to make patient-centered decisions and plan future management while addressing several well-established barriers to effective MDT-Ms.

A unique capability of IVR incorporated into this study was the ability to reconstruct patient CT imaging in 3D. Studies have shown that the use of IVR in visualization of imaging allows for greater appreciation of the relation of anatomical structures,^8^ and, in orthopedics, it has been linked with the reduction of operative time and alterations in management, resulting in fewer surgical procedures.^31^ IVR visualization of 3D models may have greater utility than the alternative of printed models, as they incorporate soft tissues that may require consideration when deciding on suitability of a fixation method.^32^

Findings of the pilot MDT displayed varied participant opinions relating to communication in IVR. The platform used in this pilot MDT is without set roles and hierarchy of IVR members, and all participants can interact with imaging, including simultaneously manipulating how 3D CT reconstructions are viewed. The absence of a clear role structure in the IVR MDT may have negatively impacted the quality of the clinical discussion. Conversely, recent literature has justified the need for a move away from a set hierarchy of roles in the medical profession.^33,34^ Despite the effect on the clinical discussion, the benefits of this feature in an IVR MDT may be increasing input from junior colleagues and medical students,^34^ which are more likely to act as bystanders in MDT-Ms rather than active involvement.^35^

The MDT-Ms in this study consisted of members of an existing orthopedic MDT to maintain relevance to true clinical practice. Cases were reviewed by two senior orthopedic surgeons with attention to clinical accuracy and points of discussion. The use of anonymized DICOMs from actual cases enabled further correlation to clinical practice and replicated surgical scenarios that would be seen in practice.

This project and its findings are not without limitations: owing to the nature of the pilot study, the small participant size limits the reliability and generalizability of the results. A greater range of health care specialties may allow improved generalizability of results to clinical practice. Further specialized teams exist in trauma and orthopedics, such as ortho-geriatrics or ortho-plastics MDTs, and there is a need to evaluate the aims of this study in these groups.^36,37^

In addition, investigating IVR MDTs in isolation may impede evaluation of efficacy and user experience, requiring the current gold standard of in-person and online MDTs for comparison. The utility of subjective metrics such as the NASA-TLX and SUS may affect result reliability. The NASA-TLX is a subjective marker of task workload, self-reported by participants, which limits the conclusions made from the results. This participant sample may also display recruitment bias in favor of individuals with positive opinions of XR technology, in contrast with the health care field where there is a reluctance to implement new technology, especially IVR.^38,39^ Finally, variations in case complexity must be addressed to ensure validity of results regarding MDT metrics, for example, the time taken per case. Tonelli et al.^40^ demonstrated the use of various markers of patient complexity in evaluating the demand of a case, which may be applied to cases prior to MDT to allow similar scope of discussion throughout the MDT.

### Further work

Our findings contribute to a novel but growing base of evidence supporting the use of IVR in MDT-Ms in clinical practice.^41,42^ Direct comparison with other MDT-M methods is needed to determine the best method of practice. A large-scale MDT trial is required, with a greater participant sample across multiple health care disciplines and comparison of IVR against in-person and online MDT-Ms. The reduced costs of IVR technology in recent years have increased accessibility while simultaneously improving capabilities, yet the uptake in health care fails to reflect this.^43^ With high cost of technology being reported as a barrier to implementation of resources, this field would benefit from an up-to-date cost analysis of IVR MDTs.^38^

## Conclusion

MDT-Ms in IVR are an emerging feasible alternative to current MDT options with several potential advantages. They enable interrogation of multiple complex datasets in a virtual realistic environment, facilitating clinical discussion. The platform developed and tested in this project addressed key barriers of attendance and enabled 3D visualization and manipulation of radiological imaging, decision-making, and surgical planning. Further development is required to test this technology at scale, integrate it into routine workflows with real patients, and further interrogate cost-effectiveness of this promising new technology.

## Abbreviations Used

2D: two-dimensional
3D: three-dimensional
BOAST: British Orthopaedic Association Standards for Trauma
CM: cephalomedullary
CT: computed tomography
DICOM: digital imaging and communications in medicine
EHR: electronic health record
Ex. Fix.: external fixation
ICE: Information Centric Engineering
IMN: intramedullary nail
IQR: interquartile range
IVR: immersive virtual reality
MDT: multidisciplinary team
MDT-M: multidisciplinary team meeting
MR: mixed reality
NASA-TLX: NASA Task Load Index
ORIF: Open Reduction and Internal Fixation
PDF: portable document format
SUS: System Usability Scale
SWOT: strengths, weaknesses, opportunities, and threats
VR: virtual reality
XR: extended reality
